# Imiquimod‐Loaded Phospholipid‐Free Small Unilamellar Vesicles Activate the Tumor Immune Microenvironment to Treat Liver Cancer and Liver Metastases

**DOI:** 10.1002/adhm.202501691

**Published:** 2025-06-17

**Authors:** Vanessa Chan, Hsin‐Mei Lee, Haruka Takata, Sheng‐Liang Cheng, Yu‐Ting Yen, Lisa Pfeifer, Luke Rushton, Po‐Han Chao, Feng Zhao, Chun Yat Ong, Nojoud Al‐Fayez, Tatsuhiro Ishida, Yunching Chen, Shyh‐Dar Li

**Affiliations:** ^1^ Faculty of Pharmaceutical Sciences University of British Columbia Vancouver BC V6T 1Z3 Canada; ^2^ Institute of Biomedical Engineering National Tsing Hua University Hsinchu 30013 Taiwan; ^3^ Department of Pharmacokinetics and Biopharmaceutics Institute of Biomedical Sciences Tokushima University 1‐78‐1, Sho‐machi Tokushima 770‐8505 Japan; ^4^ Institute of Translational Medicine and New Drug Development School of Medicine China Medical University Taichung Taiwan; ^5^ Advanced Diagnostics and Therapeutics Institute Health Sector King Abdulaziz City for Science and Technology (KACST) Riyadh 12354 Saudi Arabia; ^6^ Department of Chemistry National Tsing Hua University Hsinchu 30013 Taiwan

**Keywords:** drug delivery, imiquimod, immunotherapy, liver cancer, liver metastases, nanoparticles

## Abstract

Liver cancers are often diagnosed at advanced stages and are the fourth leading cause of cancer death globally. Liver metastases, particularly from colorectal cancer, occur in 66% of patients. Immunotherapies for these cancers are limited by immunosuppressive tumor microenvironments. To address this, phospholipid‐free small unilamellar vesicles (PFSUV) are developed to deliver the toll‐like receptor 7 agonist Imiquimod (IMQ) to hepatocytes. PFSUV consists of 83 mol% cholesterol and 17 mol% Tween80, with IMQ encapsulated in these 75‐nm particles. Intravenous administration of PFSUV‐IMQ sustained liver IFN‐*α* levels over 24 h while reducing systemic exposure. In a CT26 liver metastasis model, PFSUV‐IMQ combined with Oxaliplatin reduced tumor size, increased CD8+ T cell infiltration, and enhanced tumor apoptosis. In an HCA‐1 liver cancer model, the same treatment decreased tumor burden, increased apoptosis, and reduced lung metastases. Flow cytometry revealed increased CD86+/MHC‐II+ dendritic cells and IFN‐*γ*+ CD8+ T cells in treated tumors. RNA‐seq shows enrichment of innate immune activation genes after a single dose. These findings suggest that targeted IMQ delivery activates the tumor immune microenvironment, leading to reduced tumor burden in liver cancer and metastasis models.

## Graphical abstract

Imiquimod‐loaded nanoparticles (PFSUV‐IMQ) are found to decrease liver metastasis and liver tumor burden when administered intravenously. Flow cytometry, RNA‐seq and various experiments show that the anti‐tumor response is immune‐mediated by activating dendritic cells resulting in increased CD8 T cell infiltration and activity. Overall this article presents a novel targeted immunotherapy method for the treatment of liver cancer indications.

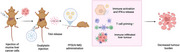

## 1 Introduction

Liver cancer is the fourth most common cause of cancer death globally.^[^ ^1^ ^]^ Hepatocellular carcinomas (HCC) account for ≈75% of all primary liver indications and are generally characterized by rapid progression and poor prognosis.^[^ ^2^ ^]^ The 5‐year standardized rate of survival is only 20% in advanced stages, where HCC Is often diagnosed.^[^ ^3^ ^]^ While the indication is typically treated with surgical resection, tumor recurrence is common at around 50–60% in literature.^[^ ^4^ ^]^ Immunotherapies have shown success with manageable toxicities in the treatment of solid tumors and exploratory trials have been performed for treatment of HCC.^[^ ^5^ ^]^ The liver is an immunologically tolerant organ, and the tumor microenvironment of HCC is highly immunosuppressive.^[^ ^6^ ^]^ These factors pose a challenge for the efficacy of currently available immunotherapies like immune checkpoint inhibitors (ICIs). Currently, combination immunotherapies like atezolizumab (anti PD‐L1) and bevacizumab (anti‐VEGF) offer a ≈35% overall response rate, leaving sufficient room for improvement.^[^ ^7^ ^]^

Beyond primary liver tumors, liver metastasis is common in other cancer indications, accounting for ≈25% of cases.^[^ ^8^ ^]^ While metastases can originate from various sources of primary tumors, colorectal cancers (CRC) are the most common. CRC is one of the most common cancers in the world and ≈66% of patients will develop liver metastasis.^[^ ^9^ ^]^ Current treatment for liver metastasis includes surgical resection or surgical resection with neoadjuvant chemotherapy. Despite these approaches, the 5‐year recurrence free survival is only ≈27%.^[^ ^10^ ^]^ ICIs have been implicated to have potential benefit in certain subsets of colorectal cancers with mismatch repair deficiency (dMMR) due to their higher mutational burden.^[^ ^11^ ^]^ Pembrolizumab (anti PD‐L1) has been demonstrated in small case studies to show complete histopathologic response in some patients with liver lesions from dMMR metastatic colorectal cancers.^[^ ^12^ ^]^ However as these subtypes of colorectal cancer only account for ≈10–20% of cases, different strategies to activate the immune system may be helpful in extending the benefit of immunotherapies.

One method to activate the immune system is using toll‐like receptors (TLRs). TLRs are pattern recognition receptors that recognize motifs of virus, bacteria or fungi, and will induce an immune response once activated.^[^ ^13^ ^]^ Imiquimod (IMQ) is an FDA‐approved small molecule from the imidazoquinolinamine class that induces the release of pro‐inflammatory cytokines through TLR7.^[^ ^14^ ^]^ As it is insoluble, IMQ is typically compounded into a cream for topical administration, and would require more sophisticated technologies to deliver it systemically.^[^ ^15^ ^]^ Additionally, excessive immune activation through TLRs can trigger cytokine storm, potentially causing multiorgan damage.^[^ ^16^ ^]^ Therefore, the ability to target the delivery of Imiquimod to the liver using nanoparticles may be a viable strategy to reverse immunosuppression in liver tumors while reducing systemic immune activation. Nanomedicines have been previously reported to be used effectively in the modulation of the tumor immune microenvironment, therefore highlighting the viability of this strategy.^[^ ^17^, ^18^ ^]^ IMQ has been reformulated into several different forms, some examples include lipophilic prodrugs for delivery to intestinal lymph nodes for oral administration, hyaluronic acid‐based nanoparticles for treatment of breast cancer, or other lipidic nanoparticles for treatment of Human Papilloma Virus.^[^ ^19^, ^20^, ^21^ ^]^ However, there are no other currently reported formulations of IMQ for the treatment of liver cancer delivered intravenously.

In this study, phospholipid‐free small unilamellar vesicles (PFSUV) were fabricated by rapid mixing an alcoholic solution of cholesterol (83 mol%) and Tween80 (17 mol%) with an aqueous buffer using a microfluidic device. IMQ was actively loaded into the aqueous core of PFSUVs. The liver‐targeted delivery and localized immune activation of PFSUV‐IMQ were compared with the free drug dissolved in DMSO. The antitumor efficacy of these two IMQ formulations was compared in one liver metastasis model and one primary liver cancer model in mice. Activation of the tumor immune microenvironment was assessed by flow cytometry, immunohistochemical staining, immunofluorescence, and RNA‐sequencing to quantify tumor infiltration of various immune effector and suppressive cells and pro‐inflammatory cytokines, respectively, to provide mechanistic insights.

## 2 Results and Discussion

### 2.1 Particles Characterization

**Figure 1:**
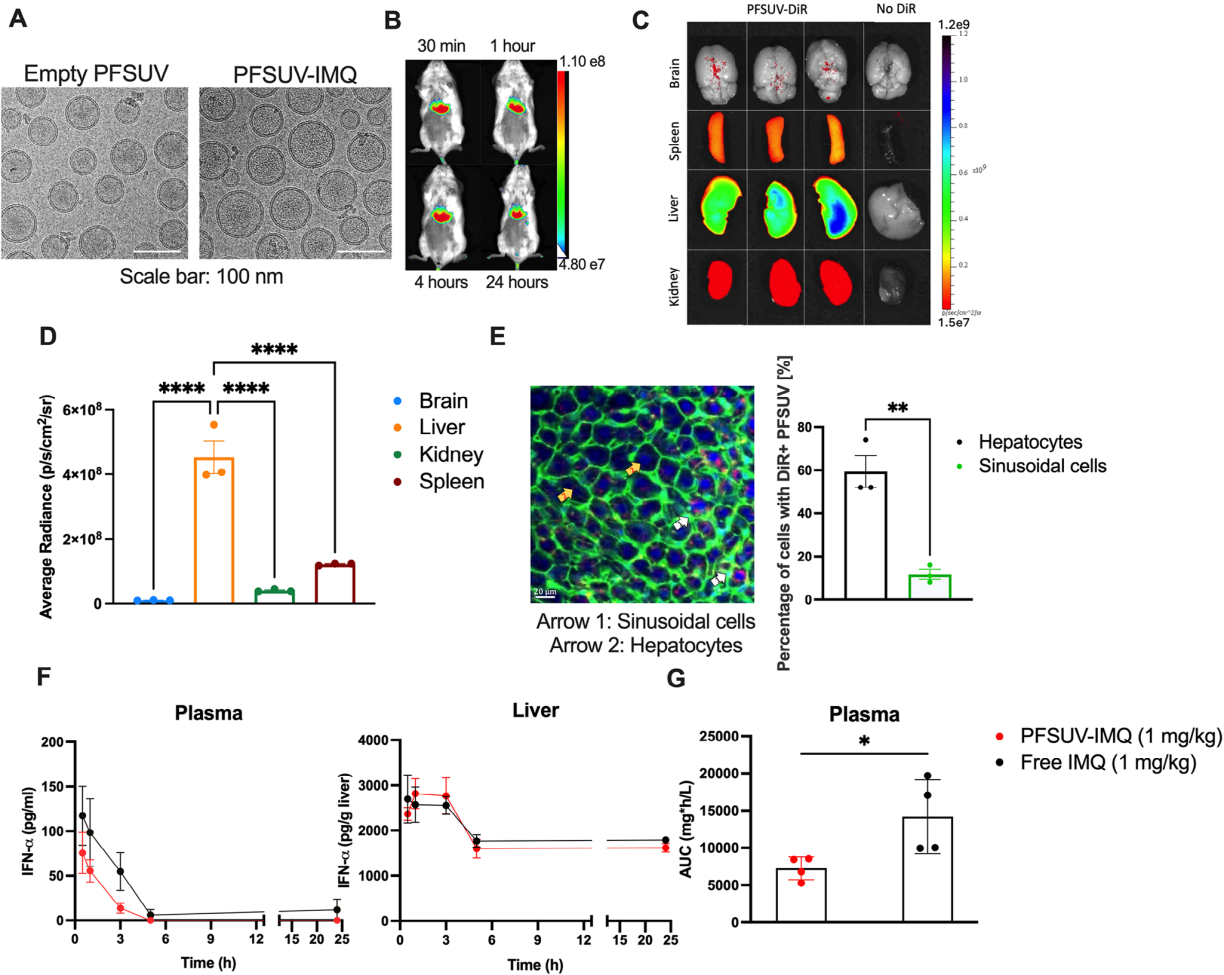
Characterization of PFSUV‐IMQ. A) Size and morphology evaluated by cryo‐transmission electron microscopy (cryo‐TEM). B) Biodistribution of DiR‐PFSUVs in mice after intravenous injection using in‐vivo imaging systems (IVIS). C) Biodistribution of brain, spleen, liver, and kidneys harvested from mice with intravenously injected DiR‐PFSUV or no DiR (B) Quantification of average radiance from different organs. E) Uptake of DiR‐PFSUVs (red) in the liver. The hepatocytes (indicated by arrows (2, yellow)) and sinusoidal cells (indicated by arrows (1)) in the liver can be differentiated by their cellular (Phalloidin staining, green) and nuclear (DAPI staining, blue) morphologies. F) Plasma and liver IFN‐*α* levels after mice were i.v. injected with PFSUV‐IMQ or free IMQ at 1 mg kg^−1^ (n = 4). G) Area under curve of plasma IFN‐*α* levels of mice given PFSUV‐IMQ or free IMQ at 1 mg kg^−1^ (n = 4). Data are presented as mean ± standard error mean (SEM). Statistics were performed using an unpaired t‐test (****, p<0.0001, **, p<0.01, *, p<0.05).

PFSUVs consisting of 83 mol% cholesterol and 17 mol% Tween80 were generated using microfluidic mixing,^[^ ^22^ ^]^ and IMQ was loaded into the particles using an ammonium sulphate gradient at a drug‐to‐lipid ratio of 1/20 (w/w) in the presence of <10% dimethyl sulfoxide (DMSO), followed by dialysis to remove DMSO and unloaded drug. Empty PFSUV were found to be 56.12 ± 0.94 nm in size with a polydispersity index (PDI) of 0.15 ± 0.02. When loaded, PFSUV‐IMQ were 75.44 ± 1.17 nm in size with a PDI of 0.166 ± 0.022, and the drug encapsulation efficiency was 98.2 ± 4.7%. The zeta potential was −3.97 ± 2.07, indicating PFSUV was a neutral particle. Particle size and morphology of PFSUVs were confirmed in cryo‐transmission electron microscopy (cryo‐TEM) (**Figure** **1**A), displaying a small unilamellar vesicular (SUV) structure. Increased electron density was visualized in the aqueous core of PFSUV‐IMQ, suggesting IMQ encapsulation. An in vitro drug release experiment in a 1:4 ratio (v/v) of PFSUV‐IMQ in fetal bovine serum at 37 °C showed stable drug retention across 2 h with a <10% drug leakage. (Figure S1, Supporting Information) This suggested IMQ was stably retained in the PFSUVs in serum for at least 2 h. PFSUVs were also found to be highly specific to the liver after intravenous (i.v.) injection. As shown in Figure 1B, within 30 min, the fluorescently tagged PFSUVs (DiR‐PFSUVs) effectively accumulated in the liver, while the uptake by other tissues was minimal. To visualize the distribution of DiR‐PFSUVs in the organs, spleen, kidney, liver, and brain were removed, fixed, and visualized under an in vivo imaging system (IVIS) one day post injection. The highest DiR signal was found in the liver with a 3.7x higher average radiance than the spleen, 11.1x higher than the kidneys and 45.4x higher average radiance than the brain. (Figure 1C,D) While the kidneys had a low signal, it was unclear as to whether this signal was from DiR‐PFSUVs or excretion of free DiR. To determine which cells in the liver were contributing to the uptake of PFSUVs, liver samples were collected 2 h post‐injection, sectioned and stained with FITC‐Phalloidin and DAPI (4,6‐diamidino‐2‐phenylindole). to differentiate hepatocytes and sinusoidal cells by their distinct nuclear and cellular morphologies. PFSUV‐DiR was found to be selectively taken up by the hepatocytes (marked by the arrows in Figure 1E), with 60% of the hepatocytes showing DiR uptake whereas only 12% of the sinusoidal cells were positive with DiR. This selective mechanism of action is unique compared to other nanoparticle formulations that are generally cleared out by sinusoidal cells, such as Kupffer cells. To investigate whether this specific targeting of PFSUV‐IMQ would result in localized immune‐stimulating effects, we administered the formulation at 1 mg kg^−1^ i.v. and found that while free IMQ and PFSUV‐IMQ induced comparable IFN‐*α* levels in the liver (Figure 1F), free IMQ provoked a twofold higher plasma exposure of IFN‐*α* (area under the curve = 14180 ± 4961 mg*h/L vs 7266 ± 1545 mg*h/L). IFN‐*α* is a potent immune‐stimulating cytokine that can turn an immunosuppressive tumor microenvironment (TME) into an immune‐active environment, but it also causes significant side effects. The data suggests a reduced safety risk of liver‐specific delivery of IMQ using PFSUV (Figure 1G). As dendritic cells are a major source of IFN‐*α* secretion and are capable of maturation after exposure to type I interferons and cytokines, we also assessed the ability of PFSUV‐IMQ to activate DC 2.4s in vitro. (Figure S2, Supporting Information) We found that PFSUV‐IMQ exposure for 24 h resulted in a significantly higher percentage of cells expressing CD80 and CD86 compared to free IMQ and other relevant controls, suggesting enhanced antigen presentation. When looking into our controls, we found that both DMSO (0.1%) and empty PFSUV showed some DC 2.4 activation. DMSO has been previously observed to induce minor levels of monocyte activation which was consistent with our findings.^[^ ^23^ ^]^ Empty PFSUVs were also found to activate the DC 2.4s, however to a lesser degree, and we suspect that this was due to the presence of Tween80 in the formulation, which has also been shown to have minor immunogenic effects.^[^ ^24^ ^]^ It should also be noted that since IMQ is only soluble in DMSO but not in water or other injectable solvents, this free IMQ formulation cannot be used clinically but only serves as a control in this research. In fact, the clinically approved IMQ is in a topical cream dosage form for treating local skin lesions, including genital warts, squamous cell carcinoma in situ, basal cell carcinoma, and actinic keratosis due to the limitations of poor solubility and selectivity. Additionally, IFN‐*α* levels were sustained throughout the 24‐h period within the liver in the PFSUV‐IMQ treated group, suggesting significant potential for immunotherapeutic efficacy.

### 2.2 Efficacy in a Model of Colorectal Cancer Metastasis to the Liver

**Figure 2:**
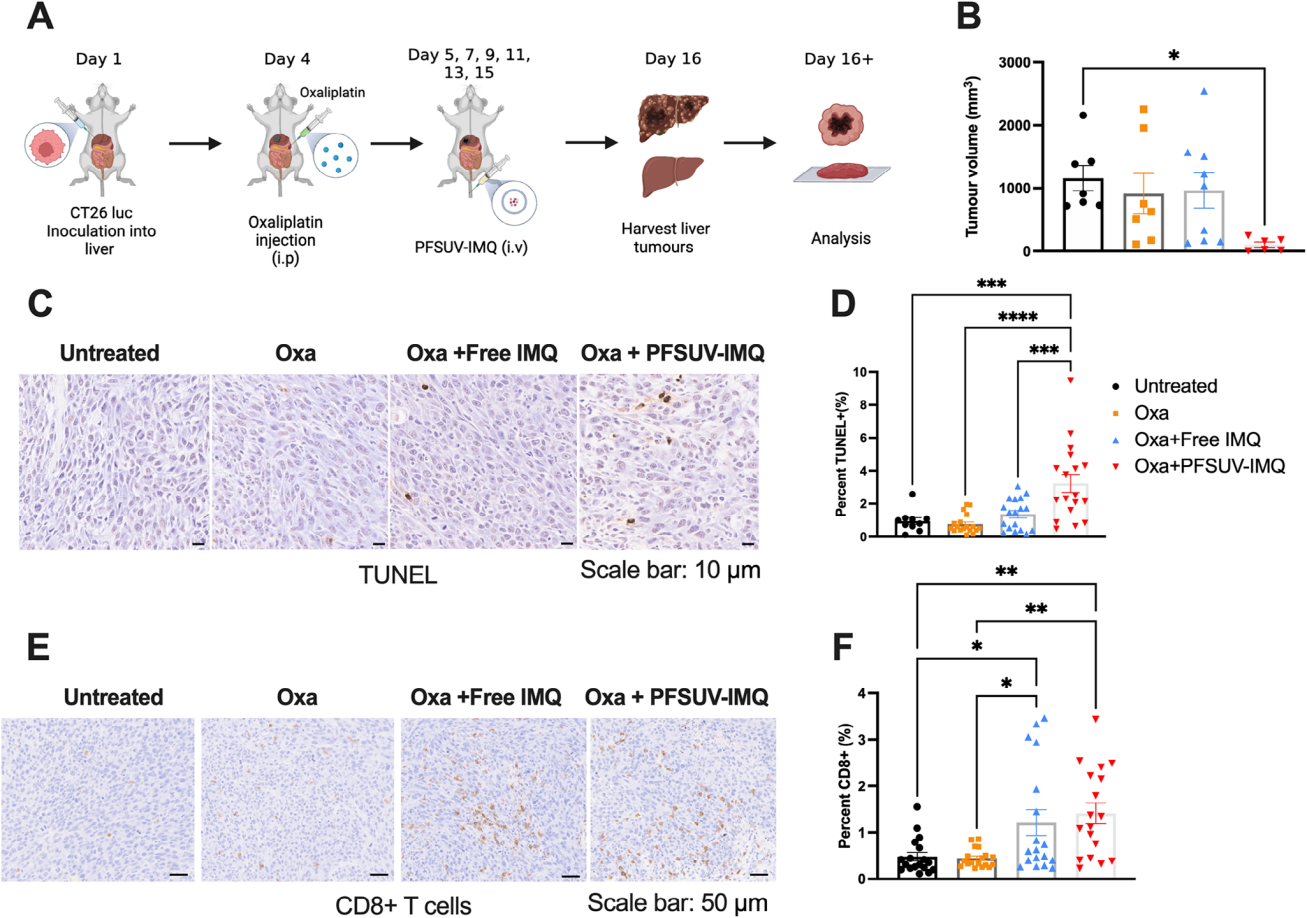
Efficacy of Oxa only, Oxa + free IMQ, and Oxa+PFSUV‐IMQ in a liver metastasis of colorectal cancer model. A) Experimental scheme. B) Tumor volumes in different treatment groups at the experimental end point. C) TUNEL staining of the harvested tumors at the experimental end point. D) Quantification of the TUNEL staining. E) Immunohistochemistry of CD8+ T cell infiltration in the tumors collected at the experimental end point. F) Quantification of CD8+ T cell infiltration into the tumors. Data are presented as mean ± standard error mean (SEM). Statistics are performed using a one‐way ANOVA with a Tukey's multiple comparisons as a post‐hoc test. (****, p<0.0001. ***, p<0.001. **, p<0.01. *, p<0.05).

To assess the efficacy of our formulation in a liver metastases model, mice were surgically inoculated with 2 × 10^5^ CT26‐luc cells in 10 µL of Matrigel into the liver. On Day 4, mice were given an i.p. injection of Oxaliplatin (Oxa) at 6 mg kg^−1^, and then PFSUV‐IMQ at 1 mg kg^−1^ was administered i.v. on Days 5, 7, 9, 11, 13, and 15 (**Figure** **2**A). Oxaliplatin (Oxa) was given intraperitoneally (i.p.). due to the challenge of administering more than 6 i.v. injections to a single mouse. On Day 16, mice were euthanized, liver tumors were harvested, and volume measurements were taken using a caliper. Tumor volumes were found to be significantly decreased in the PFSUV‐IMQ treated group compared to the control, by 11‐fold (Figure 2B). While statistical analysis did not yield significance when comparing tumor sizes from PFSUV‐IMQ to Oxa only, and free IMQ groups due to the large data variation, mean tumor sizes were decreased by 8.8‐fold compared to the Oxa only group, and 9.3‐fold compared to the Free IMQ group. Oxa only and Oxa+Free IMQ groups did not show suppressed CT26 tumor growth in the liver. Terminal deoxynucleotidyl transferase dUTP nick end labeling (TUNEL) staining showed a higher percentage of apoptotic cells in the PFSUV‐IMQ treated group (Figure 2C) compared to the Oxaliplatin only (4.2‐fold increase), free IMQ control (2.4‐fold increase), and untreated control (3.4‐fold increase) (Figure 2D). The results were comparable as that of the tumor size data in Figure 2A, showing that only Oxa+PFSUV‐IMQ induced significant apoptosis in the tumor, while the effects of other treatments, including Oxa only and Oxa+Free IMQ were insignificant. Immunohistochemical (IHC) staining revealed that the tumors of the PFSUV‐IMQ treated mice had significantly higher percentages of CD8+ T cells (Figure 2E), with a 2.9‐fold increase relative to the untreated group and 3.2‐fold increase relative to Oxa only. However, the difference between Oxa+PFSUV‐IMQ and Oxa+Free IMQ in CD8+ T cell filtration was not significant (Figure 2F). To rule out the possibility that the antitumor efficacy was partially attributed from cytotoxicity of IMQ, we examined cytotoxicity of Oxa, Oxa+PFSUV‐IMQ and Oxa+Free IMQ against CT26 and HepG2 cells. As shown in Figure S4 (Supporting Information), both Free IMQ and PFSUV‐IMQ were not cytotoxic on either cell line on their own, nor did they increase the cytotoxicity of Oxa when combined across different concentrations. This confirmed that the antitumor activity of PFSUV‐IMQ was mediated through the immune response. Taken together, these data suggest that treatment using PFSUV‐IMQ in combination with Oxa decreased the tumor burden by increasing the infiltration of CD8+ T cells and apoptotic cells in the tumor.

### 2.3 Efficacy in a Model of Primary Liver Cancer

**Figure 3:**
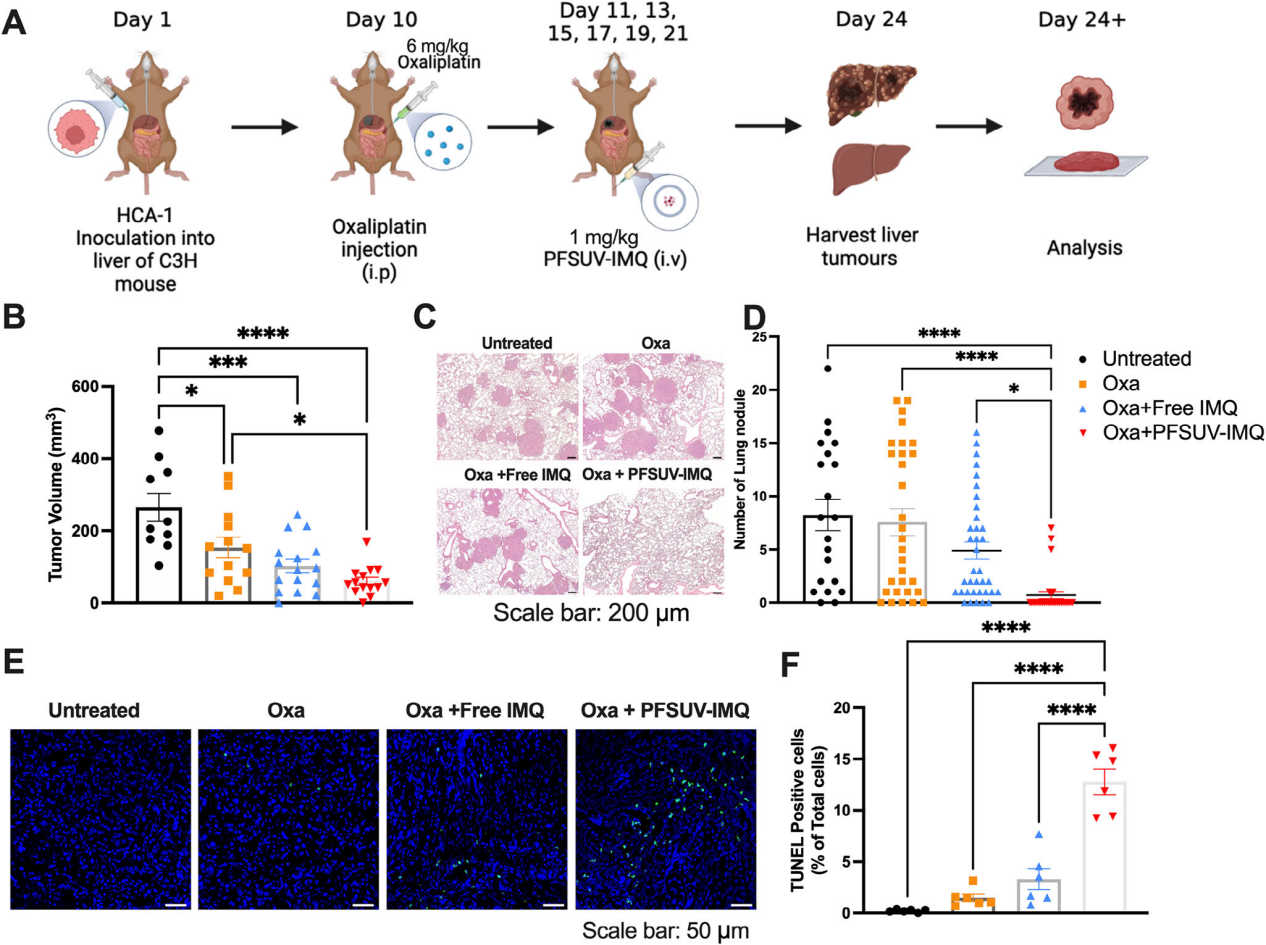
Efficacy of Oxa only, Oxa + free IMQ, and Oxa+PFSUV‐IMQ in a mouse model of primary liver cancer. A) Experimental scheme. B) Primary liver tumor volumes in different treatment groups measured using calipers on Day 24. C) Images of hemotoxylin and eosin staining of the lungs collected from mice under different treatments on Day 24. D) Quantification of tumor nodules in the lungs on Day 24. E) TUNEL staining in the liver tumors collected on Day 24. F) Quantification of TUNEL positive cells in the liver tumors collected on Day 24. Data are presented as mean ± standard error mean (SEM). Statistics are performed using a one‐way ANOVA with a Tukey's multiple comparisons as a post‐hoc test. (****, p<0.0001. ***, p<0.001. **, p<0.01. *, p<0.05).

We then tested whether PFSUV‐IMQ had anti‐cancer efficacy in a mouse model of primary liver cancer with propensity to metastasize to lungs. Control groups in this experiment contained untreated mice, Oxa only, as well as Oxa+free IMQ. To develop the model, C3H mice were inoculated with HCA‐1 liver cancer cells. After 10 days, a 6 mg kg^−1^ i.p. injection of Oxa was given to all experimental groups aside from the untreated. On Days 11, 13, 15, 17, 19, and 21, mice under the free IMQ group or PFSUV‐IMQ group received 1 mg kg^−1^ of each treatment i.v. On Day 24, mice tumors and lung tissues were harvested and processed for analysis (**Figure** **3**A). Tumor volumes on Day 24 were found to be significantly decreased in mice treated with Oxa+PFSUV‐IMQ relative to the Oxa control (2.6‐fold), and no treatment control (4.4‐fold) (Figure 3B). Although there was no statistical difference, the mean tumor volume in the Oxa+PFSUV‐IMQ group was 1.7‐fold smaller than that of the Oxa+free IMQ group. Mice treated with PFSUV‐IMQ also showed significant protection against metastatic lung nodules, whereas all other mice treated experienced metastasis. Mice treated with PFSUV‐IMQ had a 11.8‐fold, 10.8‐fold, and 7‐fold significant decrease in mean number of lung nodules compared to untreated, Oxa only and Oxa+free IMQ groups, respectively. Immunofluorescent TUNEL staining also revealed significantly more apoptotic cells in the PFSUV‐IMQ treated liver tumors relative to mice given free IMQ (3.9‐fold increase), Oxa only (8.6‐fold increase), and untreated mice (55.3‐fold increase) (Figure 3E,F). These data indicate that Oxa+PFSUV‐IMQ was more effective in inducing apoptosis in the C3H liver tumor compared to other treatments, leading to decreased primary liver tumor size and metastases to the lungs.

### 2.4 PFSUV‐IMQ Enhanced the Infiltration of Activated Dendritic Cells and Cytotoxic CD8+ T Cells into the Liver Tumors

**Figure 4:**
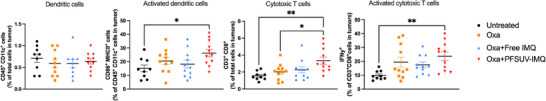
HCA‐1 tumor infiltration of dendritic cells, activated dendritic cells, CD8+ T cells and IFNγ+ CD8+ T cells after different treatments on Day 24 assessed via flow cytometry. Data are presented as mean ± standard error mean (SEM). Statistics are performed using a one‐way ANOVA with a Tukey's multiple comparisons as a post‐hoc test. (**, p<0.01. *, p<0.05).

Next, we examined changes in the immune cells infiltrating in the HCA‐1 liver tumor after different treatments described in Figure 3A. While the percentage of infiltrating dendritic cells (CD45+/CD11c+) remained relatively similar between treatment groups, a 1.7‐fold increase in the percentage of activated (CD86+, MHCII+) dendritic cells in the tumor was observed in the Oxa+PFSUV‐IMQ group, while Oxa only and Oxa+free IMQ showed no such effect (**Figure** **4**). Dendritic cells (DCs) are professional antigen‐presenting cells, and act as a linkage between the innate and adaptive immune system. Activation and increase of this population indicated that the tumor immune microenvironment (TIME) was polarizing toward a more immune‐active phenotype. The infiltrating cytotoxic T cells (CD3+, CD8+) were also significantly increased in the Oxa+PFSUV‐IMQ treated group by 1.7‐fold compared to the Oxa only group, and 2.1‐fold compared to the non‐treated group. Additionally, the percentage of activated CD8+ T cells (IFNγ+) was significantly increased by 2.4‐fold relative to the untreated control. No such TME‐priming benefit of other treatment groups, including Oxa only and Oxa+free IMQ, was found. While both T regulatory cells and CD4+ T cells were also examined through flow cytometry, no significance between groups could be observed (Figure S5, Supporting Information). By examining the types of immune cells infiltrating in the tumor, we can deduce that Oxa+PFSUV‐IMQ increased DC activation and MHC‐II presentation. This subsequently increased the percentage of infiltrating active IFNγ secreting CD8+ T cells for tumor clearance. The significant increase in CD8+ T cells was consistent with the staining observed in the IHC (Figure 2E,F). Overall, the flow cytometry data showed increased immune activation in the tumor immune microenvironment after treatment using PFSUV‐IMQ. Upregulation of CD86+/MHCII+ DCs suggested increased antigen presentation and training of cytotoxic CD8+ T cells, which was confirmed to also be significantly increased. The lack of change in the T regulatory cell population affirmed that the TIME of these liver tumors remained immunosuppressive at some level despite treatment. However, since tumor shrinkage was observed, this treatment of Oxa+PFSUV‐IMQ might be further improved by optimizing the dose or dosing regimen.

### 2.5 At a Single Dose, PFSUV‐IMQ Increased the Activation of Innate Immune Cells in the Tumor Nodules

**Figure 5:**
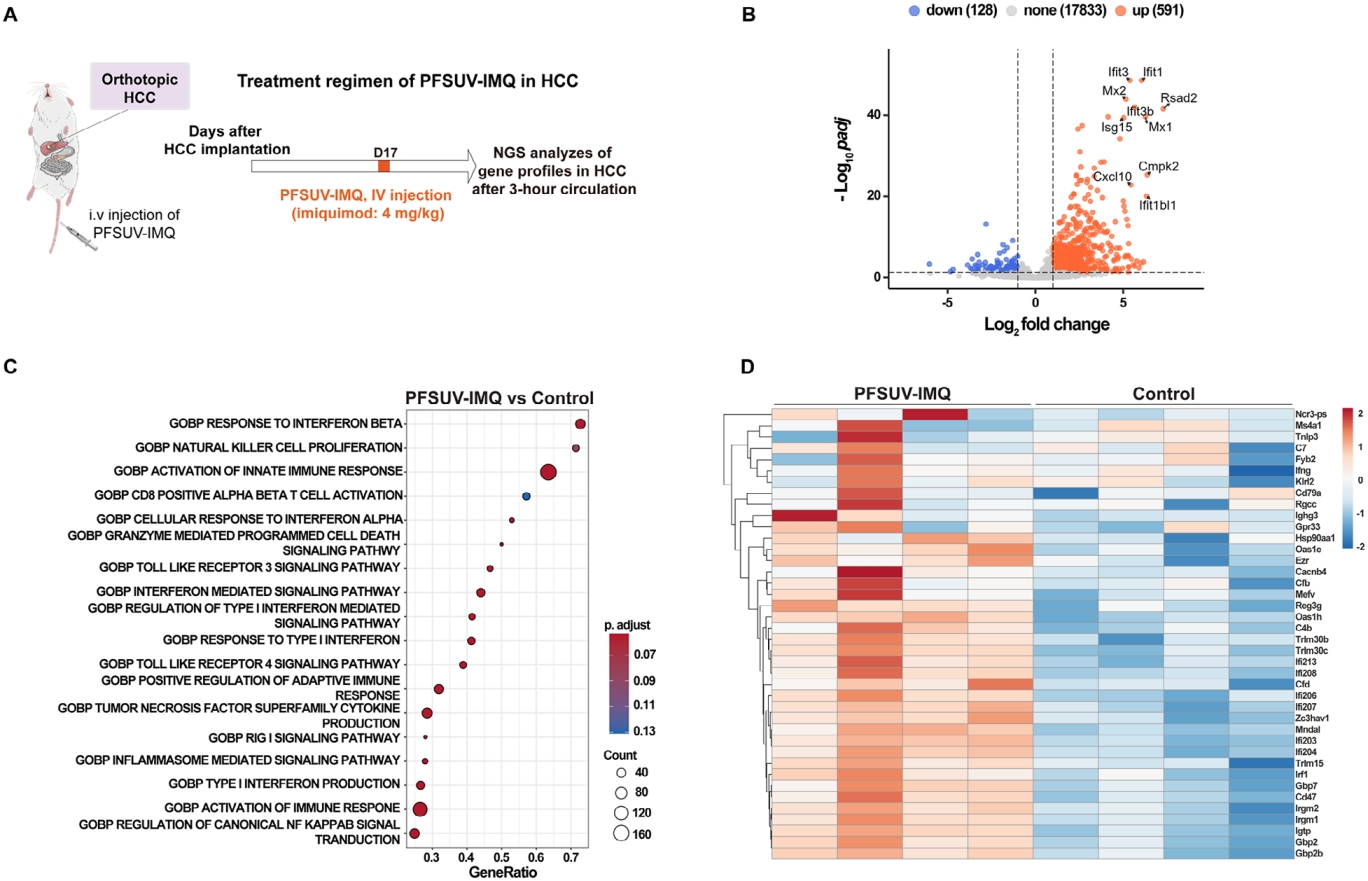
RNA‐seq data in HCA‐1 liver tumors 3 h after a single dose of PFSUV‐IMQ. A) Treatment scheme for RNA‐seq sample harvest. B) Volcano plot for upregulated and downregulated genes in the tumors treated with PFSUV‐IMQ versus untreated control. C) Gene Ontology analysis of PFSUV‐IMQ versus untreated control. D) RNA‐seq heat map.

To further our understanding of the mode of action of PFSUV‐IMQ, we performed an RNA‐seq analysis to capture the changes in the tumor immune microenvironment focusing on the immunomodulating genes. C3H mice were implanted with HCA‐1 liver tumors, and a single dose of PFSUV‐IMQ (4 mg kg^−1^) was given i.v. on day 17 after implantation. At 3 h post PFSUV‐IMQ treatment, tumors were harvested and sent for NGS analysis (**Figure** **5**A). This time point was chosen as in our IFN‐*α* analysis in the liver, we found that levels peaked at around 1 h and retained until 3 h and began to drop off after 5 h. Since IFN‐*α* is an important cytokine for early immune activation, we wanted to visualize the changes in the transcriptome prior to these levels dropping off. As shown in Figure 5B, 591 genes were found to be upregulated whereas 128 were found to be downregulated. Among the upregulated genes relative to control mice, genes related to innate immune activation such as *Ifit1, Ifit3, Mx1 and Mx2, Ifit3b, Isg15, Cxcl10, Cmpk2, Ifit1bl1* and *Rsad2* were identified. *Ifit1* and *Ifit3* expression is often associated with anti‐viral responses, and all other genes are typically upregulated in response to interferons. As IMQ activates TLR7, a sensor for viral infection, such gene upregulation suggested successful delivery of IMQ to the tumor using PFSUV. Biological processes that were found to be enriched through Gene Ontology (GO) analysis included response to Interferon beta, natural killer cell proliferation and activation of innate immune responses (Figure 5C). Natural killer cells are typically found in the tumor when MHC Class Is are downregulated, which is a common immune evasion technique. Enrichment of this pathway after administration suggests that the selected time point was early in the deployment of an immune response against the tumor. While CD8 positive alpha beta T cell activation was also enriched, the 3‐h time point was likely also too early to assess for a significant adaptive immune response. IMQ is known to induce the release of interferon alpha through TLR7 and the MyD88 pathway, so enrichment of this pathway was also expected. Both TLR3 and TLR4 signalling pathways were also found to be increased in this analysis. While IMQ is a TLR7 agonist, it is possible that the overall inflammation generated by its presence played a role in the release of antigens that triggered TLR3 and TLR4 activation. The activation of these TLR pathways could release factors that suppress cancer proliferation and attract T and natural killer cells to enhance the overall anticancer effect.^[^ ^25^ ^]^ NF‐κB signal transduction, retinoic acid inducible gene (RIG‐1), and inflammasome mediated signaling pathways also supported the hypothesis of an activated immune response. The function of RIG‐1 lays in sensing viral RNAs to trigger an inflammatory response through release of type I interferons and cytokines.^[^ ^26^ ^]^ RIG‐1 is also a positive regulator for NF‐κB signalling, which could subsequently contribute to the inflammasome activation pathway enrichment. Taken together, these pathway enrichment clusters suggested that PFSUV‐IMQ stimulated TLRs that activated NF‐κB signaling, resulting in transcription of type I interferons and cytokines like TNF‐*α*. These possibly resulted in inflammasome development and priming of an adaptive immune response. While the timepoint was likely too early to properly assess for an adaptive immune response, CD8 positive alpha beta T cell activation pathways and granzyme mediated programmed cell death signaling were slightly enriched, suggesting there was an increase in granzyme‐secreting CD8+ T cells. All Gene Ontology Biological Processes (GOBP) pathways are summarized in **Table** **1**. These results were well correlated to the increased tumor infiltration of activated DCs and CD8+ T cells shown in Figure 4. After administration of PFSUV‐IMQ, innate immune activation pathways were found to be significantly upregulated in the RNA‐seq, which matched the increase in IFN‐*α* that was observed in the liver (Figure 1F). As dendritic cells were hypothesized to be the main drivers behind the type 1 interferon secretion and were found to be enriched in tumors after drug administration, we validated the activation of DC 2.4s by PFSUV‐IMQ in cell culture. As shown in Figure S2 (Supporting Information), increased DC 2.4s were found to be expressing CD80 and CD86 when treated with PFSUV‐IMQ, suggesting this treatment was effective in enhancing the antigen presenting ability of these cells through TLR7 activation. Altogether, TLR7 signaling started when the TLRs sensed IMQ, which then triggered a cascade through the MyD88 dependent pathway, resulting in increased secretion of interferons and cytokines. TLR7 activation also resulted in upregulation of DC maturation proteins like CD80/CD86.^[^ ^27^ ^]^ As CD80/CD86 are co‐stimulatory molecules that activate CD28 on cytotoxic T cells, these activated DCs could present antigen to CD8+ T cells, thus activating them (IFN‐y+) for tumor‐specific killing.^[^ ^28^ ^]^ (Figure 4) It is important to note that the mechanism of PFSUV‐IMQ differs from existing immunotherapies like immune checkpoint inhibitors (ICIs) as PFSUV‐IMQ activates DCs in the tumor to present antigen to CD8+ T cells. Based on the RNA‐seq, we observe a modulation of the tumor immune microenvironment from an immunosuppressive phenotype to an immune‐active phenotype. Currently available ICIs only focus on blocking the negative interaction between T cells and cancer cells, therefore, we are introducing a novel mechanism of immunotherapy through PFSUV‐IMQ. Overall, NGS analysis showed an increased expression of early inflammatory genes in treated mouse tumors relative to the untreated control. The results were consistent with the flow cytometry data, where dendritic cells were found activated after PFSUV‐IMQ treatment. Taken together, these data suggested PFSUV‐IMQ was effective in tuning the tumor immune microenvironment from immunosuppressive to immune active, thus facilitating the overall decrease in tumor volume. Since the immunological mechanisms of PFSUV‐IMQ differ from those of approved immunotherapies, such as ICIs, this novel therapy may synergize with existing drugs to enhance efficacy.

**Table 1:** The Gene Ontology Biological Processes (GOBP) pathway analysis revealed differential expression of innate and adaptive immune regulatory pathways in murine HCA‐1 tumors treated with PFSUV‐IMQ compared to the untreated.

Name of gene set	ES	NES	pvalue
GOBP_RESPONSE_TO_INTERFERON_BETA	0.9087	2.6543	0.0012
GOBP_RESPONSE_TO_TYPE_I_INTERFERON	0.8576	2.4934	0.0012
GOBP_INTERFERON_MEDIATED_SIGNALING_PATHWAY	0.8136	2.4398	0.0012
GOBP_REGULATION_OF_TYPE_I_INTERFERON_MEDIATED_SIGNALING_PATHWAY	0.8685	2.3850	0.0013
GOBP_ACTIVATION_OF_INNATE_IMMUNE_RESPONSE	0.6914	2.3659	0.0010
GOBP_TYPE_I_INTERFERON_PRODUCTION	0.7247	2.3309	0.0011
GOBP_CELLULAR_RESPONSE_TO_INTERFERON_ALPHA	0.9221	2.1508	0.0015
GOBP_TOLL_LIKE_RECEPTOR_4_SIGNALING_PATHWAY	0.7127	2.0462	0.0012
GOBP_TOLL_LIKE_RECEPTOR_3_SIGNALING_PATHWAY	0.7830	2.0342	0.0014
GOBP_REGULATION_OF_CANONICAL_NF_KAPPAB_SIGNAL_TRANSDUCTION	0.5922	1.9885	0.0010
GOBP_ACTIVATION_OF_IMMUNE_RESPONSE	0.5684	1.9856	0.0010
GOBP_RIG_I_SIGNALING_PATHWAY	0.7769	1.9464	0.0014
GOBP_INFLAMMASOME_MEDIATED_SIGNALING_PATHWAY	0.6966	1.9309	0.0013
GOBP_TUMOR_NECROSIS_FACTOR_SUPERFAMILY_CYTOKINE_PRODUCTION	0.5638	1.8934	0.0010
GOBP_GRANZYME_MEDIATED_PROGRAMMED_CELL_DEATH_SIGNALING_PATHWAY	0.8407	1.8801	0.0015
GOBP_NATURAL_KILLER_CELL_PROLIFERATION	0.7224	1.8417	0.0029
GOBP_POSITIVE_REGULATION_OF_ADAPTIVE_IMMUNE_RESPONSE	0.5268	1.7146	0.0011
GOBP_CD8_POSITIVE_ALPHA_BETA_T_CELL_ACTIVATION	0.6159	1.700	0.006

## 3 Conclusion

In summary, PFSUVs were loaded with IMQ, a typically insoluble TLR7 agonist for systemic administration. This formulation showed strong liver and hepatocyte selectivity and differentially activate the immune response in the liver. Intravenous PFSUV‐IMQ administration resulted in sustained IFN‐*α* levels in the liver with significantly lower plasma levels compared to free IMQ. When tested in combination with Oxa chemotherapy in a mouse model of liver metastasis from colorectal cancer, tumors were found to be significantly reduced in volume with increased apoptotic cells and infiltrating CD8+ T cells. In a mouse model of liver cancer, PFSUV‐IMQ combination with Oxa also showed decreases in tumor burdens, as well as superior control for lung metastasis. Flow cytometry and RNA‐seq of tumors after PFSUV‐IMQ administration revealed a significant increase in activated DCs and activated CD8+ T cells in the tumor as well as elevated gene expression indicative of innate immune activation. Overall, systemic delivery of IMQ using PFSUVs demonstrated specific tuning of the TIME and reduced liver tumor burdens.

## 4 Experimental Section

### Phospholipid‐Free Small Unilamellar Vesicles (PFSUV) Preparation and Characterization

PFSUVs containing cholesterol and Tween80 were prepared as previously described.^[^ ^22^ ^]^ Cholesterol and Tween80 (5:1 molar ratio) were dissolved in ethanol (10 mg total lipid/mL) and mixed with 250 mM ammonium sulfate at a flow ratio of 1:3 and a total flow rate of 20 mL min^−1^ at the room temperature using NanoAssemblr Benchtop (Precision Nanosystems, Vancouver, BC, Canada). The particles were collected and dialyzed overnight against 100 mM sodium acetate buffer (pH 5) to remove the ethanol. Particle size, PDI and zeta potential of the PFSUVs were measured by dynamic light scattering using Zetasizer NanoZS (Malvern Instruments, Malvern, UK).

### Imiquimod (IMQ) Loading

IMQ was actively loaded into PFSUV using the solvent‐assisted loading technique (SALT).^[^ ^29^ ^]^ IMQ was dissolved in DMSO and mixed with PFSUV at a 1/5 (w/w) drug‐to‐lipid ratio. The final concentration of DMSO in the mixture was <10% (v/v). The mixture was incubated at 37 °C for 1 h and quenched on ice for 2 min. The formulation was further dialyzed against HEPES (4‐(2‐hydroxyethyl)‐1‐piperazineethanesulfonic acid) buffered saline (HBS, pH 7.4) overnight. For Ultra High‐Performance Liquid Chromatography (UPLC) measurements, 15 µL of the PFSUVs were mixed with 45 µL ethanol and sonicated for 5 min, followed by injection of 10 µL of the sample for analysis.

### Cryo‐TEM

Empty PFSUVs and PFSUV‐IMQ (25 mg lipid/mL) were added to a glow‐charged copper grid using an FEI Mark IV Vitrobot (FEI, Hillsboro, OR, USA). Imaging was done using a 200 kV Glacios microscope equipped with a Falcon III camera at the UBC High Resolution Macromolecular Cryo‐Electron Microscopy facility (Vancouver, BC, Canada).

### Ultra Performance Liquid Chromatography (UPLC)

IMQ concentration was measured using an ACQUITY UPLC H‐Class System (Waters, Milford, MA) coupled online to a photodiode array (PDA) detector (wavelength 320 nm). A BEH‐C18 column (inner diameter: 2.1 mm; length: 100 mm; particle size: 1.7 m, Waters) was used for separation with a gradient mobile phase containing a mixture of eluent A and B (0.1% aqueous TFA and 0.1% TFA in methanol, respectively) at a flow rate of 0.3 mL min^−1^. The gradient used was as follows: 0 min: A/B (95/5); 2 min: A/B (95/5); 8 min: A/B (0/100); 11 min: A/B (0/100); 11.1 min: A/B (95/5); 13 min: A/B (95/5). Lipid concentrations were determined using the same chromatographic method in conjunction with an evaporative light scattering (ELS) detector.

### Mice

For tissue distribution and cytokine stimulation studies, female CD‐1 mice (18–20 g, 5–6 weeks old) were purchased from The Jackson Laboratory (Bar Harbor, ME). These in vivo studies were conducted in accordance with an established protocol (A22‐0141) approved by the Animal Care Committee of the University of British Columbia (Vancouver, BC, Canada). For the mouse model of liver metastasis from a colorectal cancer origin, male BALB/c mice (4‐5 weeks old, 18–20 g) were purchased from Japan SLC (Shizuoka, Japan). All animal experiments were conducted with the approval of the Animal and Ethics Review Committee of Tokushima University (Approved No. T2022–36). For the primary liver cancer model studies, male C3H/HeNCrNarl (4–6 weeks old, 25 g) mice were purchased from the National Laboratory Animal Center (Taipei, Taiwan). All animals received humane care, in compliance with the “Guide for the Care and Use of Laboratory Animals” published by the National Academy of Sciences. All animal experiments were conducted in accordance with an established protocol (Protocol #109074), approved by the Animal Research Committee of National Tsing Hua University.

### Cytokine Stimulation in the Liver

PFSUV‐IMQ was i.v. administered at a dose of 1 mg kg^−1^ into female CD‐1 mice. Blood and liver were collected at 30 min, 1 h, 3 h, 5 h and 24 h. After collection, blood was transferred into EDTA(ethylenediaminetriacetic acid)‐coated tubes and separated into plasma via centrifugation for 10 min at 500 x g at 4 °C. Mouse liver was collected after euthanasia and washed twice with PBS, blotted dry, and weighed (≈0.5 g) into a 1.5 mL microtube (Next Advance, Inc., Troy, NY, USA). Protease inhibitor cocktail in PBS (1:100 v/v dilution, Sigma Aldrich) was added to the tissue (0.3 mL per 0.1 g tissue), and homogenized at 4 °C for 5 min using a tissue homogenizer Bullet blender (Next Advance, Inc., Troy, NY, USA) at an instrument speed of 10. Samples were then subjected to ELISA analysis for IFN‐*α* levels according to the manufacturer's protocols (Thermofisher, Catalog # BMS6027).

### Biodistribution

Female CD‐1 mice (18–20 g, 5–6 weeks old) purchased from The Jackson Laboratory (Bar Harbor, ME) were i.v. injected with DiR‐PFSUVs at a dosage of 0.3 µg g^−1^. Mice were imaged using an IVIS Imaging System (Caliper Life Sciences, Waltham, MA) under nose‐cone administered isoflurane anesthesia at 30 min, 1 h, 4 h and 24 h. For organ biodistribution, female CD‐1 mice (31‐39 g, 18–20 weeks old) were purchased from Charles River Laboratories (Senneville, QC, Canada). DiR‐PFSUVs were injected at a dosage of 0.3 µg g^−1^ and one day post injection, mice were euthanized, and brain, liver, kidneys and spleen were harvested, washed with PBS and stored in 10% (v/v) formalin in PBS overnight at room temperature. The organs were then imaged using the same IVIS Imaging System. Average radiance was quantified using the Living Image 3.1 Software (Caliper Life Sciences, Waltham, MA).

### Intrahepatic Co‐Localization

PFSUV‐DiR was i.v. injected into mice at a dosage of 0.3 µg DiR/g. They were then euthanized 2 h post injection and the liver was harvested, washed with phosphate‐buffered saline (PBS) and then stored in 10% (v/v) formalin in PBS at room temperature overnight. 40 µm tissue sections were then prepared from the fixed livers using a vibratome (Precisionary Instruments LLC, Boston, MA). These sections were added to PBS and incubated in 0.1% (v/v) Triton X‐100 in PBS for 5 min, before being washed three times in PBS and then incubated in 1% (v/v) bovine serum albumin in PBS For 10 min. Tissue sections were washed 3 times with PBS again and then incubated in Alexa Fluor 488 Phalloidin (APh, 80 µL, 1 U mL^−1^). To remove excess staining, sections were further washed with PBS and then mounted on a glass slide with a drop of Fluoroshield containing DAPI (Sigma–Aldrich). A confocal microscope (Zeiss LSM 700) at 20x magnification and analyzed with ZEN software (both Carl Zeiss, Oberkochen, Germany).

### Efficacy in a Model of Liver Metastases from a Colorectal Cancer Origin

To develop the liver metastasis model, mice were placed in the supine position, and a 1‐ to 1.5‐cm skin incision was made in the upper abdominal wall, followed by a 1‐cm incision in the peritoneum to expose the liver. CT26‐Luc cells 2.0 × 10^5^ in 10 µl of 2:1 RPMI 1640/Matrigel (Corning, NY, USA)] were gently injected under the surface of the left lobe of the liver. The insertion site was sealed with an absorbable hemostatic material (SURGICEL, Johnson and Johnson, New Brunswick, NJ, USA). The liver was returned within the body after the injection, and the abdominal incision was closed in 2 layers with 4‐0 silk surgical thread (Natsume Seisakusho, Tokyo, Japan). After 4 days, mice were injected intraperitoneally (i.p.) with 6 mg kg^−1^ of Oxaliplatin. On days 5, 7, 9, 11, 13, and 15, mice were i.v. injected with either 1 mg kg^−1^ PFSUV‐IMQ, 1 mg kg^−1^ free IMQ, or saline. Throughout the experiment, tumor growth was monitored using luminescence where 100 µL of 15 mg mL^−1^ D‐luciferin potassium salt (FUJIFILM Wako Pure Chemical Corporation, Osaka, Japan) was injected i.p. and then mice were imaged on IVIS (Xenogen, CA, USA). Mice were sacrificed on Day 16 and tumors were collected and volume measurements were taken. Tumors were then fixed in 10% formalin (v/v) in PBS. Paraffin‐embedded liver section, IHC staining for CD8+ T cells, TUNEL staining, and microscopic imaging were performed by Wax‐it Histology Services Inc. (Vancouver, BC, Canada). Image analysis and quantification was done using ImageJ.

### Efficacy in a Model of Primary Liver Cancer

The primary liver cancer model was developed as described.^[^ ^30^ ^]^ After 10 days, mice were i.p. injected with 6 mg kg^−1^ of Oxa. On days 11, 13, 15, 17, 19, and 21, mice were i.v. injected with either 1 mg kg^−1^ PFSUV‐IMQ, 1 mg kg^−1^ free IMQ or saline. Mice were sacrificed on Day 24 and tumors, and lungs were collected. Volume measurements were taken of the liver tumors. Lung tissue was cut into small pieces and fixed in 4% PFA (in PBS) overnight before being embedded in paraffin wax. The sections were then stained with immunofluorescent dyes and observed with an inverted microscope (IX83, Olympus, Japan).

### Flow Cytometry Analysis

To evaluate the immune profile in the orthotopic HCC model, mice at the end point from the above efficacy study were perfused with PBS via intracardiac injection, followed by euthanasia. Tumors were enzymatically digested at 37 °C for 30 min using collagenase type 1A (1.5 mg mL^−1^) and hyaluronidase (1.5 mg mL^−1^) prepared in RPMI medium. The resulting cell suspensions were stained with specific antibodies for flow cytometry analysis. The antibodies employed included CD45‐FITC (no. 30‐F11), 7‐AAD, CD11c‐APC (no. 550261), CD86‐PE‐Cy7 (no. 560582), CD3e‐APC (no. 145‐2C11), CD8‐PE‐Cy7 (no. 53–6.7), CD16/CD32 BD Fc Block (no. 2.4G2), all procured from BD Biosciences (CA); MHCII‐APC‐eflour780 (no. 47‐5321‐82) from eBioscience (California, USA).

To detect IFN‐*γ* expression in CD8 T cells, the cell suspensions were fixed with 4% paraformaldehyde, permeabilized using Cytofix/Cytoperm solution (BD Biosciences), and stained intracellularly with IFN‐*γ*‐APC‐Cy7 (no. 561479, BD Biosciences, California, USA) in accordance with the manufacturer's instructions. The immune profile of tumor samples was performed using a BD FACSAria III flow cytometer and data were analyzed using FACSDivaTM software. The gating strategy for the flow cytometry data is shown in Figure S3 (Supporting Information).

### Next‐Generation Sequencing Gene Expression Analysis

C3H mice were orthotopically implanted with the HCA‐1 tumor as previously described. On day 17, mice were either given saline or 4 mg kg^−1^ PFSUV‐IMQ at a single dose i.v. After 3 h, mice were euthanized and their tumors taken for analysis. RNA samples were extracted from the treatment and control groups using the RNeasy Kit (Qiagen), and the integrity of the samples was assessed with the RNA Nano6000 assay kit (Agilent Technologies, CA, USA). Library preparation and sequencing was conducted by Biotools Co., Ltd. The resulting FASTQ files were aligned to the reference genome using TopHat v2.0.12, and gene‐level read counts were quantified with HTSeq v0.6.1. Gene expression levels were determined as FPKM (Fragments Per Kilobase of transcript per Million mapped reads) based on gene length and mapped read counts.

RNA sequencing‐derived read counts were used to calculate gene expression. Normalization and differential expression analysis were performed using edgeR (v3.28.1) and DESeq2 (v1.26.0), respectively. Differentially expressed genes (DEGs) were identified with edgeR using a p‐value and false discovery rate (FDR) threshold of <0.05. Heatmaps of differential expression were visualized via the ClusVis web server, and gene set enrichment analysis (GSEA) was applied to interpret biological variability. The processed data were submitted to the Gene Expression Omnibus (GEO) repository with the accession number GSE289163 (GEO release date, March 1^st^, 2025).

### Statistical Analysis

All data were presented as mean ± SEM. Statistical analysis was performed with GraphPad Prism version 8.0 (GraphPad Software, San Diego, CA, USA). Comparisons between groups were made by unpaired t‐test and one way ANOVA. A difference with p < 0.05 was considered to be statistically significant.

### Animal Ethics

All animals received humane care, in compliance with the “Guide for the Care and Use of Laboratory Animals” published by the National Academy of Sciences. All in vivo studies were conducted in accordance with either an established protocol (A22‐0141) approved by the Animal Care Committee of the University of British Columbia (Vancouver, BC, Canada), animal protocol (No. T2022‐36) approved by the Animal and Ethics Review Committee of Tokushima University, or animal protocol (Protocol #109074) approved by the Animal Research Committee of National Tsing Hua University

## Conflict of Interest

The authors declare no conflict of interest.

## Supporting information



Supporting Information

## Data Availability

The data that support the findings of this study are available from the corresponding author upon reasonable request.
