# Supplementary material for: Imiquimod‐Loaded Phospholipid‐Free Small Unilamellar Vesicles Activate the Tumor Immune Microenvironment to Treat Liver Cancer and Liver Metastases
Source: Adv Healthc Mater. 2025 Jun 17;14(22):2501691. doi: 10.1002/adhm.202501691 (PMC12391647; doi:10.1002/adhm.202501691)
Supplement: Supplementary file 1 — Supporting Information [file ADHM-14-0-s001.docx]

## Additional Materials and Methods

### DC 2.4 activation

DC 2.4 were plated at 5x10^4^ cells per well in a 24-well plate and left to grow overnight in RPMI1640 media supplemented with 10% FBS, 1X non-essential amino acids and 1X penicillin and streptomycin. The media was replaced the next day and PFSUV-IMQ, and free IMQ were added at 25 ug/mL per well (100 μL). DMSO (0.1%) and empty PFSUV controls were added accordingly and incubated for 24 hours. Cells were then removed from the 24-well plate by using 0.25% trypsin, then replated into 96-well V-bottom plates for flow cytometry analysis. Cells were stained with CD11c- PE Vio770 (REA759), CD80-FITC (REA983) and CD86-PE (REA1190) from Miltenyi Biotec (Bergisch Gladbach, Germany).

### In vitro drug retention

An in vitro drug retention study in serum was done to evaluate the amount of IMQ retained in the PFSUVs in plasma circulation prior to reaching the liver. PFSUV-IMQ was incubated at a 1:4 ratio (v/v) in FBS at 37°C. Since the formulation was shown to effectively accumulate in the liver after 2 hours, the drug retention was examined at 10, 30 and 120 minutes. At each time point, samples were collected and purified via size exclusion chromatography (SEC) to remove the released IMQ. Ultra performance liquid chromatography (UPLC) was then used to measure the retained concentration of IMQ.

### Cytotoxicity assay

To assess the cytotoxicity of Oxa and PFSUV-IMQ *in vitro,* HepG2 cells and CT26 cells were plated onto 96-well plates and incubated for 24 hours (7500 cells/well for CT26 and 10000 cells/well for HepG2). Either Oxa alone, OXA + PFSUV-IMQ or Oxa + free IMQ was added to the cells and incubated for 24 hours prior to addition of 25μL of XTT sodium salt (1 mg/mL) activated by N-methyl dibenzopyrazine methyl sulfate (PMS) (25 μM) (MedChemExpress, NJ, USA). Plates were then incubated for 4 hours and then measured for absorbance (450 nm and 650 nm as the reference wavelength) on the BioTek Synergy Mx. (BioTek, VT, USA)


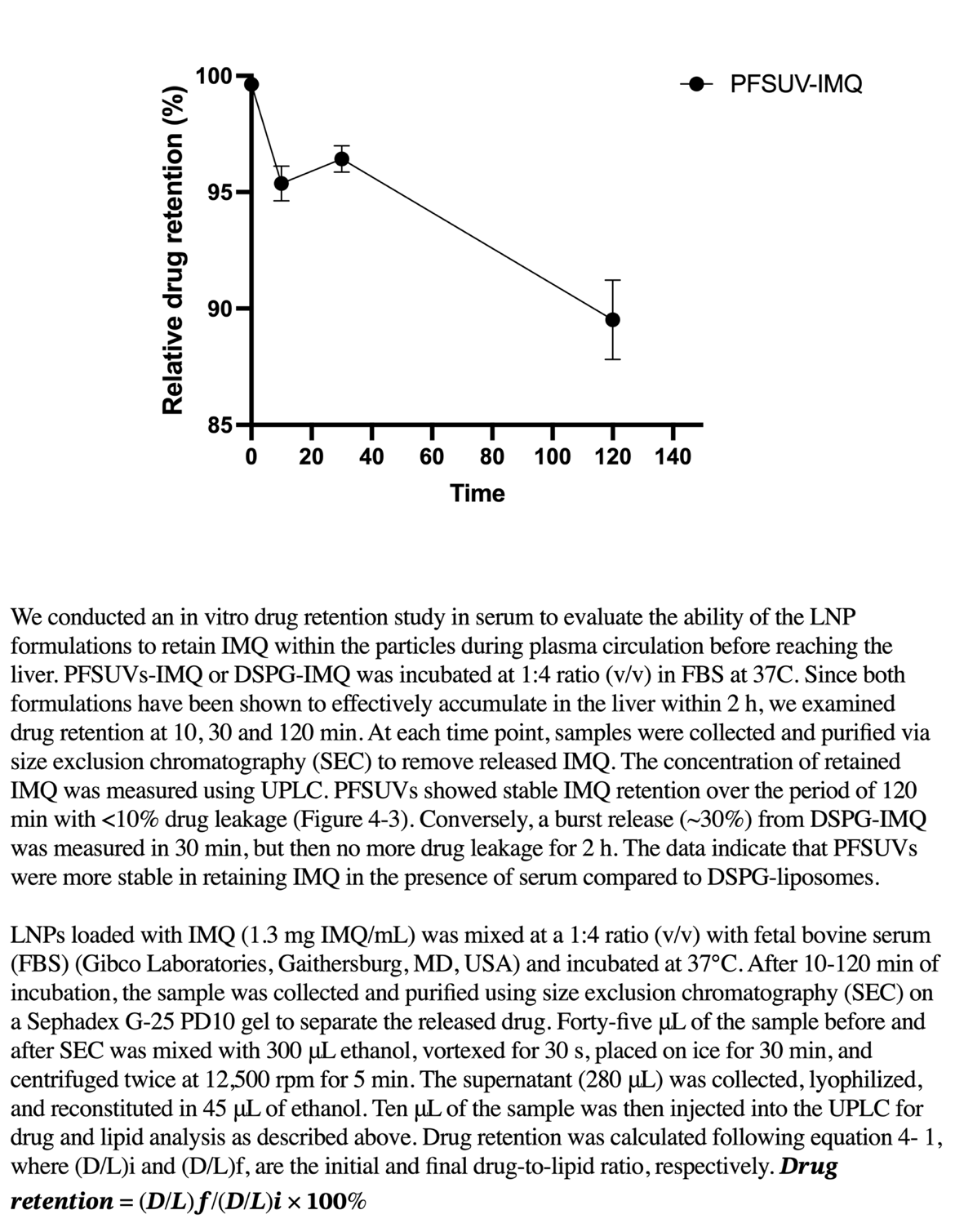


Figure S1. In vitro drug retention of PFSUV-IMQ in 25% fetal bovine serum (FBS) at 37°C. Data= mean ± SD (n=3).
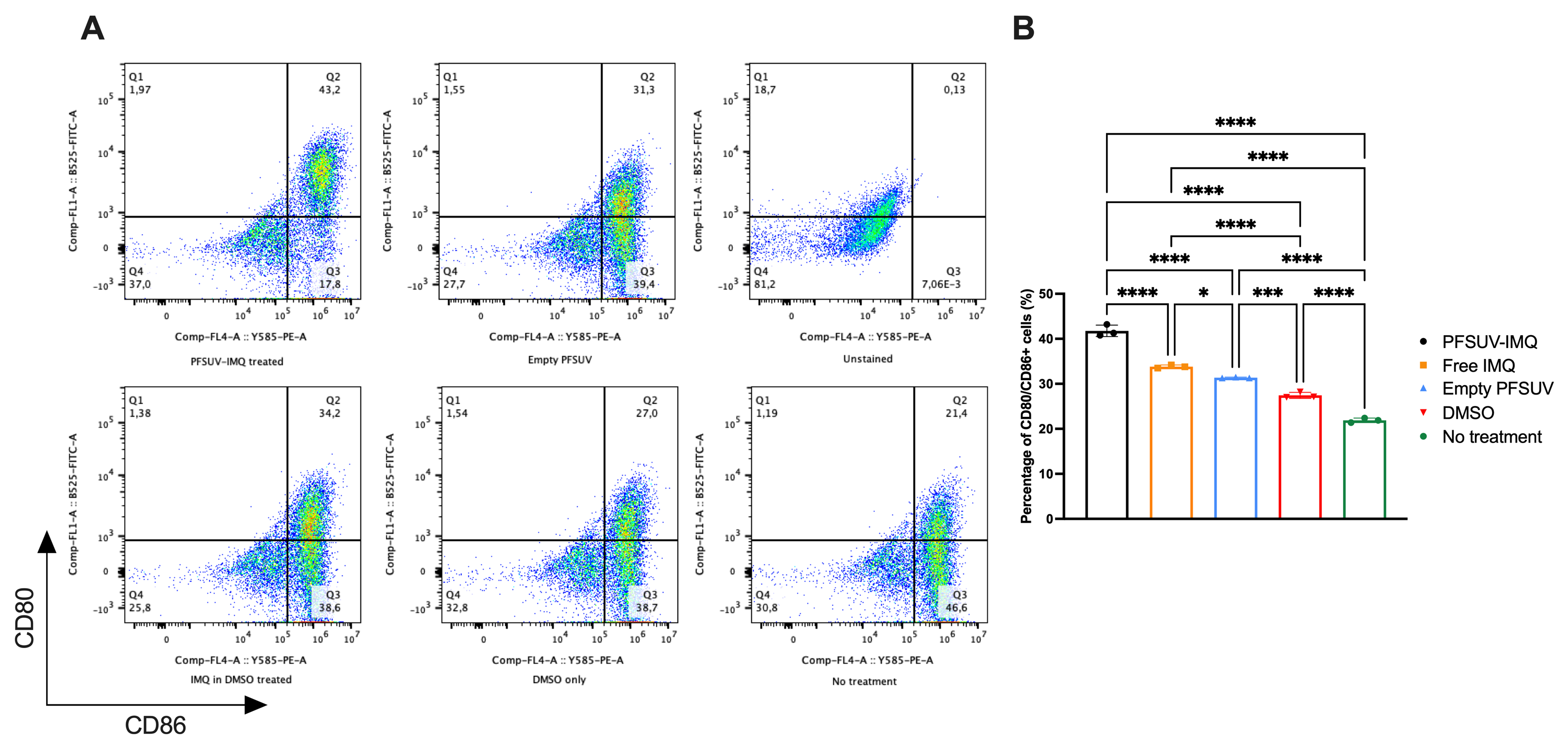


Figure S2. DC 2.4 activation across different treatments. (A) Flow plots for CD80+/CD86+ DC 2.4s. (B) Quantification of CD80+/86+ cells across different treatments. (****, p<0.0001. ***, p<0.001. **, p<0.01. *, p<0.05) Data=mean ± SD (n=3). Statistics are performed using a one-way ANOVA with a Tukey’s multiple comparisons as a post-hoc test.

Figure S3. Gating strategy of flow plots. (A) Gating of CD8 and CD4 T cells. (B) Gating of DC activation. (C) Gating of T cell activation.


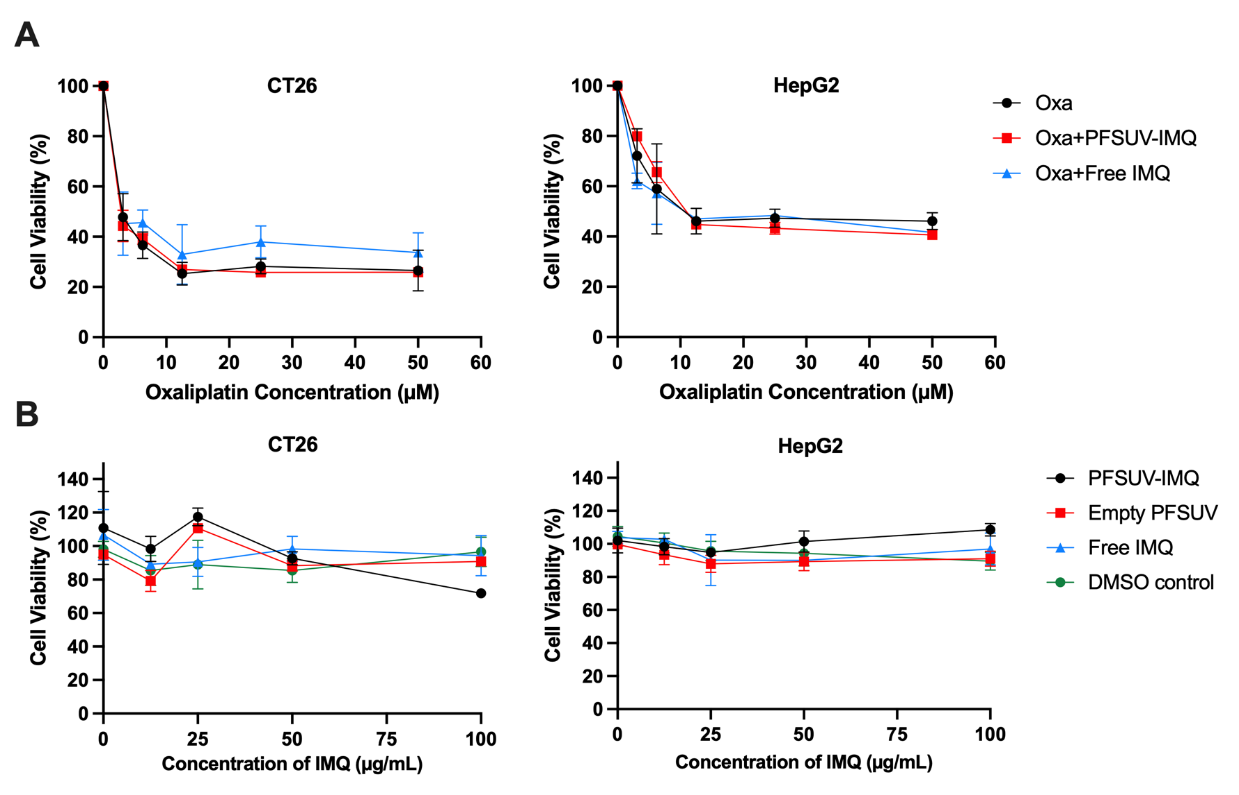


Figure S4. CT26 and HepG2 cell viability measured by XTT after exposure to treatments and their combinations. (A) Cell viability after exposure to treatments. Either Oxa alone, Oxa + PFSUV-IMQ or Oxa + free IMQ was added to the cells and incubated for 24 hours before XTT analysis. Oxa concentration was varied at 50 µM, 25 µM, 12.5 µM, 6.25 µM and 3.125 µM. IMQ concentration was fixed at 25 µg/mL. (B) Cell viability after exposure to IMQ treatments. PFSUV-IMQ, and free IMQ were added at varying concentrations (100, 50, 25, 12.5 µg IMQ/mL) Cells were incubated for 24 hours before XTT analysis. Data=mean ± SD (n=3). Statistics are performed using a one-way ANOVA with a Tukey’s multiple comparisons as a post-hoc test.

Figure S5. HCA-1 tumour infiltration of CD4+ T cells, Treg cells, Ki67+ cells, and Granzyme B+ cells after different treatments on Day 24 assessed via flow cytometry. Data are presented as mean±SEM. Statistics are performed using a one-way ANOVA with a Tukey’s multiple comparisons as a post-hoc test.
